# Evaluation of the effect of *Matricaria recutita* monotherapy or in combination with photodynamic therapy on tissue repair in the dorsum of the tongue of rats*

**DOI:** 10.1590/1678-7757-2023-0211

**Published:** 2023-10-27

**Authors:** Juliana Borges de Lima DANTAS, Tila FORTUNA, Hortência Resende DELLA CELLA, Fábio Luís Meneses de Sousa da SILVA, Rejane Conceição SANTANA, Gabriela Botelho MARTINS

**Affiliations:** 1 Universidade Federal da Bahia Instituto de Ciências da Saúde Programa de Pós-graduação em Processos Interativos dos Órgãos e Sistemas Salvador Bahia Brasil Universidade Federal da Bahia, Instituto de Ciências da Saúde, Programa de Pós-graduação em Processos Interativos dos Órgãos e Sistemas, Salvador, Bahia, Brasil.; 2 Faculdade Adventista da Bahia Laboratório de Histologia e Embriologia Cachoeira Bahia Brasil Faculdade Adventista da Bahia, Laboratório de Histologia e Embriologia, Cachoeira, Bahia, Brasil.; 3 Universidade Federal da Bahia Instituto Multidisciplinar de Reabilitação e Saúde Salvador Bahia Brasil Universidade Federal da Bahia, Instituto Multidisciplinar de Reabilitação e Saúde, Salvador, Bahia, Brasil.

**Keywords:** Chamomile, Matricaria, Photochemotherapy, Methylene blue, Wound healing

## Abstract

The search for treatments that accelerate the healing of lesions is of constant interest. *Matricaria recutita* (chamomile) is a plant with antimicrobial, anti-inflammatory, and healing properties, and antimicrobial Photodynamic Therapy (aPDT) eradicates microorganisms, which favors tissue repair.

**Objective:**

This study aimed to evaluate the effect of the topical use of chamomile with or without aPDT on tissue repair in rats’ tongues.

**Methodology:**

A total of 75 male Wistar rats underwent standardized ulceration on the dorsum of the tongue using a punch of 5 mm diameter and were randomly allocated into the following groups: control (G1), chamomile fluid extract (G2), chamomile infusion (G3), aPDT (G4), and chamomile infusion + aPDT (G5). On the 3^rd^, 7^th^, and 14^th^ days postoperatively, euthanasia was performed, and the ulcers were measured using calipers. The presence of edema, inflammatory infiltrate, cellularity, re-epithelialization, and characterization of total collagen were evaluated using sections stained with Hematoxylin and Eosin and Red Sirius. Histomorphometry analyses of the percentage of total collagen, the distance from the basal layer to the epithelial surface, and the thickness of the stratum corneum were performed. Descriptive (absolute/relative frequencies and modes) and exploratory analyses were performed. The associations between the groups and the presence of ulcers were analyzed with Fisher’s exact test. All analyses were performed using the R program and statistical significance was set at p=0.05.

**Results:**

The G2 positively modulated the exudative and proliferative phases of repair, both clinically (*p*<0.0001) and histologically, whether in descriptive or inferential analyses (*p*<0.05). The G3 showed a significant difference in clinical parameters compared with G1 (*p*<0.0001). The G4 and G5 did not positively modulate tissue repair.

**Conclusion:**

The chamomile fluid extract showed better outcomes for tissue repair in the rat tongue.

## Introduction

Ulcers are the most common oral cavity lesions. They are characterized by pain and discomfort owing to the loss of epithelium and exposure of nerve endings in the underlying connective tissue, thus affecting individuals’ quality of life.^1,2^ Due to their varied etiopathogenesis, their treatment is often complex, especially when these lesions become infected.^3^

Furthermore, several therapeutic options, which can be used alone or in combination, can heal these ulcers by modifying the repair phases.^4-7^ Among the main resources used to accelerate tissue repair, synthetic agents, including antimicrobials and immunomodulators, stand out beyond laser photobiomodulation, which includes a device with stimulated emission of non-ionizing radiation.^6^ In recent decades, the increasing use of medicinal herbs has become frequent due to several advantages, such as easy access, low cost, and minimal adverse effects.^5,7^

Chamomile, also known as *Matricaria recutita*, is one of the most used infusions in the world. It is a natural medicinal plant belonging to the Asteraceae family widely known and highly available to the public, and has anti-inflammatory, antimicrobial, antispasmodic, antioxidant, sedative, and healing properties.^5,7^ It has approximately 120 bioactive components, of which phenolic compounds stand out and are responsible for the antioxidant action,^8^ including flavonoids and their derivatives (apigenin, luteolin, patulenin, and quercetin), α-bisabolol, related sesquiterpenes, and chamazulenes.^9^ Flavonoid compounds, chamazulenes, and sesquiterpenes are responsible for its anti-inflammatory and antimicrobial activities. In addition, flavonoids inhibit histamine release, and α-bisabolol promotes granulation tissue formation during wound healing.^1,9,10^ Studies have shown that this agent can accelerate wound closure, since it acts in all stages of the tissue repair process.^1,4,5^

Antimicrobial Photodynamic Therapy (aPDT) is used to reduce and/or eliminate microorganisms with the interaction of a photosensitizer dye and a light source with an appropriate wavelength under oxygenated tissue, forming reactive oxygen species (ROS) and photo-oxidating viruses, bacteria, parasites, and fungi.^11,12^ In Dentistry, aPDT has proven effective in treating recurrent herpes labialis lesions^13^ and periodontal disease^14^ and decontaminating dental implants.^15^ Despite being a potent therapy for eradicating microorganisms, which contributes indirectly to the tissue repair process, the benefits of aPDT as a healing modulator agent are not well elucidated in the literature, and the few studies on this approach have shown contradictory results.^16-19^

Despite the promising results of chamomile and aPDT for tissue repair, no studies have addressed the combination of these agents and if their action are potentiated or modified after the combination. The hypothesis of this study is that both therapies promote positive modulation of the tissue repair process, and the null hypothesis tested is that the groups tested show no difference regarding the modulation of tissue repair process. Therefore, this study aimed to evaluate the effect of chamomile with or without aPDT on tissue repair in surgical wounds on the dorsum of the tongue of rats.

## Methodology

### Experimental study using an animal model

This study was approved by the Committee on Ethics in the Use of Animals of the Health Sciences Institute of the Federal University of Bahia (UFBA) under protocol number 3562050618. This experimental laboratory study used an animal model, including 75 male *Wistar* rats *(Rattus norvegicus albinus*; weighing 200-230 g). The animals were housed in specific plastic cages in groups of five, with good lighting conditions (light/dark cycle of 12/12 h) under a temperature of ± 24–26°C), and were fed a commercial diet (Nuvilab, Quimtia, Colombo-PR, Brazil) and water *ad libitum* throughout the experiment. All animals underwent a 7-day acclimatization period in a vivarium environment.

### Surgical procedure

All animals were weighed using a standard scale (Toledo, São Bernardo do Campo-SP, Brazil) and were sequentially anesthetized with an intraperitoneal injection of 10% Ketamine Hydrochloride mixture (90 mg/kg; Ketamina^®^, Farex do Brasil Ltda., Porto Alegre, Brazil) and 2% Xylazine Hydrochloride (5 mg/kg; Xilazin^®^, Syntec, São Paulo, Brazil). The surgical procedure was performed on day 0 (D0) of the experiment, in which standardized ulceration was performed in the center of the dorsum of the tongue of the rats using a circular punch scalpel (Biopsy Punch, Stiefel, Germany) of 5 mm diameter, limited to the mucosa, without muscular involvement. The ulcer was measured using a 150 mm analog caliper (Zaas, Florianópolis, Brazil) by a single evaluator, previously calibrated.

### Experimental groups

After performing the surgical procedure on D0, the animals were randomly allocated by lottery to five experimental groups containing 15 animals each. Then, the first dose of each therapy was applied on D0 immediately after surgery, as described below.

Control Group (G1): Daily application of saline solution (0.9%) using a flexible plastic rod with cotton on the ends (Cotonetes, Johnson & Johnson, São Paulo, Brazil) every 12 h.

Fluid Extract Group (G2): Daily topical application of 10% chamomile fluid extract (Ad-Muc^®^, Biolab, São Paulo, Brazil) by using a flexible plastic rod with cotton at the ends every 12 h, following the manufacturer’s instructions. Composition of Ad-Muc^®^: 100 mg of fluid extract of *Chamomilla recutita* (L.) Rauschert for each gram of ointment. Other ingredients: glycerol, lanolin alcohols, cetostearyl alcohol, white soft sodium petrolatum, xanthan gum, methylparaben, peppermint essence, myrrh tincture, mineral oil, sodium saccharin, and purified water.^20^

Chamomile Infusion Group (G3): Daily topical application of chamomile infusion (Chás Real^®^, Paraná, Brazil) every 8 h by simulating mouthwash using a dropper, in which an average of five drops, totaling 0.25 mL of infusion, was poured on the back of the tongue. The content was obtained by infusing one sachet submerged in 250 mL of boiling water for 3 minutes, according to the manufacturer’s instructions. Subsequently, it was cooled to a temperature of 20–22°C and stored for approximately 24 h.

aPDT Group (G4): Topical application of 0.01% methylene blue dye (Chimiolux; Hydro-Farma, Belo Horizonte, Minas Gerais, Brazil) for 3 minutes using a flexible rod, followed by irradiation using a Low Power Laser (Laser DMC^®^ Therapy EC, São Carlos, São Paulo, Brazil), in continuous mode, with power of 100 mW, wavelength of 660 nm, output area of 0.028 cm^2^, energy density of 117.85 J/cm^2^, energy in the center of the ulcer of 3.3 J, and application of 33 s, according to the protocol proposed by Sperandio, et al.^18^ (2010), applied on alternate days.

Chamomile Infusion and aPDT Group (G5): Daily topical application of chamomile infusion every 8 h combined with aPDT performed on alternate days in the morning shift, as described above for the isolated groups. A chamomile infusion was administered after aPDT was performed.

To apply the modulating agents, the animals were restrained using an atraumatic plastic device^21^, which reduced their stress. In addition, in all groups in which chamomile, aPDT, and saline solution was administered, the animals were kept without water or food for 30 min.

### Clinical evaluation

Macroscopic characteristics of the repair process of ulcers were analyzed daily in the morning using ambient light, wooden spatulas, and elastic ties to help the animals open their mouths. This evaluation was performed using two classifications: present, when clinical signs of the wound were observed; and absent, when the ulcer was absent, and the repair process had been completed macroscopically.

On the day of euthanasia, visual inspection was performed using the same analog caliper used on D0, with the diameter of the wound recorded in mm after euthanasia, when still present, and the measurement was performed by a single evaluator, previously calibrated.

### Euthanasia of animals

The animals were euthanized in the morning by randomizing and administering an injectable anesthetic of 10% Ketamine Hydrochloride combined with 2% Xylazine Hydrochloride using the technique of anesthetic deepening with intraperitoneal administration at a ratio of 90:10 mg/kg. The established periods of death were 3, 7, and 14 days after surgery.

### Histological processing

After confirming death, specimens of the animals’ tongues were obtained by autopsy using a scalpel blade (No. 15; Descarpack, Santa Catarina, Brazil) and Goldman fox scissors (Golgran, São Paulo, Brazil). Then, the fragment was fixed in 4% buffered formalin (pH 7.4) and was sent for routine histological processing at the Laboratory of Histology and Embryology of Bahia Adventist College in Cachoeira-Bahia, to obtain slides stained with Hematoxylin and Eosin (HE) and Sirius Red.

### Morphological and morphometric evaluations

Histological analysis of sections stained with HE was performed blindly and in triplicate, with a minimum interval of one week between them, by a previously calibrated examiner. The presence of edema, inflammatory infiltrate, and fibroblastic cellularity was evaluated semi-quantitatively and coded with scores categorized as intense (+++), moderate (++), mild (+), and absent (0), as described by Medrado, et al.^22^ (2008).

In the same sections, the characteristic predominance of the inflammatory infiltrate type, categorized as polymorphonuclear or monomorphonuclear, was evaluated in triplicate, as well as the degree of re-epithelialization, classified as absent, partial, completely disorganized, or completely organized. In the latter case, the presence of epithelium with all layers and the differentiation of the lingual papillae were considered.

In the morphological analysis of the sections stained with Sirius Red, the deposition pattern and thickness of the collagen fibers were evaluated: whether thick or delicate; whether the stretching pattern was organized or disorganized; and whether the depth of their deposition at the wound site was normal or increased.

Quantitative morphometric analysis of the percentage of total collagen area to the total area of the tissue was performed with ImageJ Software (National Institutes of Health, Bethesda, MD, USA), standardization of a 720,000 µm^2^ area of the image, considering height of 800 µm and length of 900 µm, and was carried out by two blinded examiners, with previous calibration, also performed from three distinct areas of tissue sections from the two edges and center of the ulcer, stained with Sirius Red, in the 10× objective.

### Statistical analysis

A database was created in Microsoft Excel 2016 for descriptive analysis to identify the general and specific characteristics of the studied samples, including quantitative analysis of the percentage of collagen.

Data on morphological variables were presented as absolute and relative frequencies and modes.

Inter-rater reproducibility analyses were performed for the percentage of collagen area and linear morphometric measurements using a paired t-test and intraclass correlation analysis, respectively. First, calculating the intraclass correlation coefficient (ICC), considering coefficients <0.40 as poor, 0.40 to <0.75 as satisfactory, and ≥0.75 as excellent, then, the averages of the two evaluators for these variables.

Descriptive and exploratory analyses of the data were performed. Linear models and the interaction between them were used considering group and time effects to analyze the percentage of collagen area. A mixed generalized linear model for repeated measures over time was used for the clinically assess the ulcer. The associations between the groups and ulcers were analyzed using Fisher’s exact test. All analyses were performed using the R program (Foundation for Statistical Computing, Vienna, Austria), and statistical significance was set at *p*=0.05.

## Results

### Clinical analysis

Table 1 shows clinical data on the number of animals with ulcers and their respective percentages in each experimental group over time. From the 8^th^ to the 12^th^ day, the groups (*p*<0.05) showed a significant difference regarding the percentage of animals with ulcers. The healing process was faster for the two chamomile formulations (G2 and G3), with complete regression of the ulcers on the 9^th^ day, followed by G5, with complete healing on the 11^th^ day. The aPDT group (G4) obtained the complete repair on the 12^th^ day, and G1 showed healing of the ulcers on the 14^th^ day.

**Table 1:** Number of animals with ulcer and total number of animals in the group (%), in each group over time (LBO/UFBA, 2022)

Dia	G1	G2	G3	G4	G5	p-value
D0	15/15 (100.0%)	15/15 (100.0%)	15/15 (100.0%)	15/15 (100.0%)	15/15 (100.0%)	-
D1	15/15 (100.0%)	15/15 (100.0%)	15/15 (100.0%)	15/15 (100.0%)	15/15 (100.0%)	-
D2	15/15 (100.0%)	15/15 (100.0%)	15/15 (100.0%)	15/15 (100.0%)	15/15 (100.0%)	-
D3	15/15 (100.0%)	15/15 (100.0%)	15/15 (100.0%)	15/15 (100.0%)	15/15 (100.0%)	-
D4	10/10 (100.0%)	10/10 (100.0%)	8/10 (80.0%)	10/10 (100.0%)	10/10 (100.0%)	0.1412
D5	10/10 (100.0%)	10/10 (100.0%)	8/10 (80.0%)	10/10 (100.0%)	10/10 (100.0%)	0.1412
D6	10/10 (100.0%)	10/10 (100.0%)	7/10 (70.0%)	10/10 (100.0%)	10/10 (100.0%)	0.0230
D7	10/10 (100.0%)	9/10 (90.0%)	7/10 (70.0%)	9/10 (90.0%)	10/10 (100.0%)	0.2011
D8	5/5 (100.0%)	3/5 (60.0%)	1/5 (20.0%)	5/5 (100.0%)	5/5 (100.0%)	0.0048
D9	5/5 (100.0%)	0/5 (0.0%)	0/5 (0.0%)	4/5 (80.0%)	5/5 (100.0%)	<0.0001
D10	5/5 (100.0%)	0/5 (0.0%)	0/5 (0.0%)	2/5 (40.0%)	1/5 (20.0%)	0.0011
D11	4/5 (80.0%)	0/5 (0.0%)	0/5 (0.0%)	1/5 (20.0%)	0/5 (0.0%)	0.0146
D12	3/5 (60.0%)	0/5 (0.0%)	0/5 (0.0%)	0/5 (0.0%)	0/5 (0.0%)	0.0154
D13	2/5 (40.0%)	0/5 (0.0%)	0/5 (0.0%)	0/5 (0.0%)	0/5 (0.0%)	0.1231
D14	0/5 (0.0%)	0/5 (0.0%)	0/5 (0.0%)	0/5 (0.0%)	0/5 (0.0%)	-

Fisher Exact Test. Source: own authorship.

Regarding the size of the surgical wound, Table 2 shows that at the initial time (D0), ulcer measurement was not significantly different (*p*>0.05) between the groups, which confirms the expected standardization in this period. At three days, the size of the ulcer showed a greater reduction in the G4 group (aPDT) compared with the other groups (*p*<0.05). On day 7, all groups showed a significant reduction of the ulcer compared with day 3. Furthermore, in this same period, the ulcer measurement was significantly higher in G1 than in G4 (*p*<0.05). On day 14, no groups exhibited ulcers (*p*>0.05).

**Table 2:** Mean (standard deviation) of the measurement in millimeters of the initial and final ulcer, according to the group (LBO/UFBA, 2022)

Measurement	Group	Time (days)		
		3 days	7 days	14 days
Initial	G1	9.60 (0.89)^Ab^	9.20 (0.84)^Aa^	9.40 (1,14)^Aa^
	G2	10.40 (0.55)^Aa^	9.60 (0.89)^Ba^	9.17 (0,41)^Ba^
	G3	9.20 (0.45)^Ab^	8.90 (0.55)^Aa^	9.10 (0,22)^Aa^
	G4	9.20 (0.45)^Ab^	9.20 (1.10)^Aa^	9.20 (0,84)^Aa^
	G5	9.60 (0.55)^Ab^	8.80 (0.84)^Aa^	9.60 (0,89)^Aa^
Final	G1	*7.10 (0.89)^Aa^	*5.60 (0.55)^Ba^	*0.00 (0.00)^Ca^
	G2	*6.80 (0.45)^Aa^	*4.20 (2.49)^Bab^	*0.00 (0.00)^Ca^
	G3	*8.00 (1.58)^Aa^	*4.10 (2.46)^Bab^	*0.00 (0.00)^Ca^
	G4	*5.60 (0.22)^Ab^	*3.60 (2.10)^Bb^	*0.00 (0.00)^Ca^
	G5	*7.80 (0.45)^Aa^	*4.50 (1.12)^Bab^	*0.00 (0.00)^Ca^

* It differs significantly from the initial time (p≤0.05). Different letters (uppercase comparing the times horizontally and lowercase comparing the groups vertically within each measurement) indicate statistically significant differences (p≤0.05). p(time)<0.0001; p(group)=0.1531; p(measurement)<0.0001; p(time x group)=0.0757; p(measurement x group)=0.0417; p(time x measurement)<0.0001; p(time x group x measurement)=0.0061. Analysis performed with generalized mixed linear model. Source: own authorship

### Histomorphological analysis

**Figure 1:**
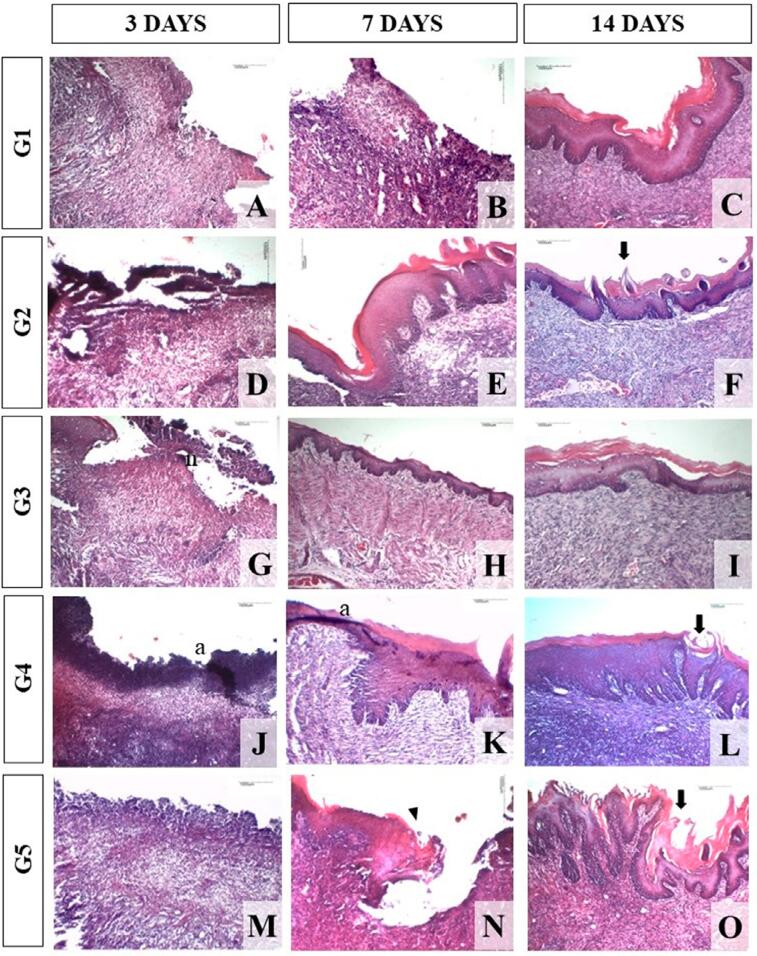
Morphological analysis of experimental groups. Photomicrographs of histological sections stained with Hematoxylin and Eosin (HE) obtained in the 10× objective of the centers of the ulcers, demonstrating the degrees of re-epithelialization in the groups within 3, 7, and 14 days. Notably, the sections of all groups showed no re-epithelialization on the 3rd day. On the 7th day, only G2 exhibited complete re-epithelialization of all sections. The other groups exhibited initial epithelium formation at the wound edges, as shown in the G5 section (arrowhead). On day 14, although all groups showed complete epithelial coverage, G4 and G5 presented sections with no re-epithelialization. The G2, G4, and G5 also showed differentiation of lingual papillae (arrow). a: technique artifact. n: necrosis (LBO/UFBA, 2022)

**Figure 2:**
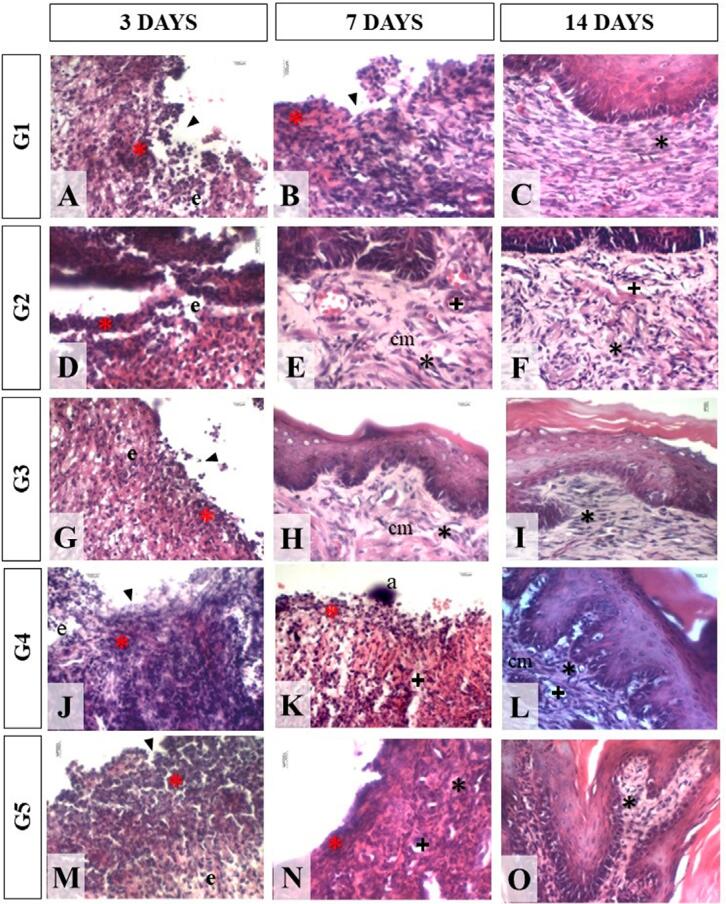
Morphological analysis of experimental groups. Photomicrographs of histological sections stained with HE obtained in the 40× objective of the regions of the ulcers, illustrating the histomorphological characteristics in the groups within 3, 7, and 14 days. Notably, on the 3rd day, all groups exhibited edema (e) and inflammatory infiltrate (red asterisk). Ulcer area (arrowhead) can be observed in this period as well. At 7 days, G2 and G3 showed tissue cellularity – fibroblasts (black asterisk) and a collagen matrix (cm). On day 14, most groups showed no ulcer in all sections (G1, G2, and G3), with complete re-epithelialization. a: technique artifact. Black cross: blood vessel light (LBO/UFBO, 2022)

Table 3 shows the results of the morphological analysis of the sections stained with HE, and Figures 1 and 2 show the histological characteristics observed in the groups.

**Table 3:** Frequency distribution of the results of morphological analyses according to the group and time of sacrifice (LBO/UFBA, 2022)

Variable	Results	G1			G2			G3			G4			G5		
		3 days	7 days	14 days	3 days	7 days	14 days	3 days	7 days	14 days	3 days	7 days	14 days	3 days	7 days	14 days
		n=5	n=5	n=5	n=5	n=4	n=5	n=4	n=5	n=5	n=5	n=5	n=5	n=5	n=5	n=5
Edema	Absent	0(0.0%)	3(60.0%)	5(100%)	0(0.0%)	4(100%)	5(100%)	0(0.0%)	1(20.0%)	5(100%)	1(20.0%)	1(20.0%)	4(80.0%)	0(0.0%)	3(60.0%)	4(80.0%)
	Discreet	0(0.0%)	2(40.0%)	0(0.0%)	0(0.0%)	0(0.0%)	0(0.0%)	0(0.0%)	3(60.0%)	0(0.0%)	0(0.0%)	2(40.0%)	0(0.0%)	0(0.0%)	1(20.0%)	0(0.0%)
	Moderate	0(0.0%)	0(0.0%)	0(0.0%)	3(60.0%)	0(0.0%)	0(0.0%)	1(25.0%)	1(20.0%)	0(0.0%)	0(0.0%)	0(0.0%)	0(0.0%)	0(0.0%)	0(0.0%)	0(0.0%)
	Intense	5(100%)	0(0,0%)	0(0.0%)	2(40.0%)	0(0.0%)	0(0.0%)	3(75.0%)	0(0.0%)	0(0.0%)	4(80.0%)	2(40.0%)	1(20.0%)	5(100%)	1(20.0%)	1(20.0%)
Inflamma-tory infiltrate	Absent	0(0.0%)	0(0.0%)	1(20.0%)	0(0.0%)	4(100%)	2(40.0%)	0(0.0%)	0(0.0%)	2(40.0%)	1(20.0%)	1(20.0%)	1(20.0%)	0(0.0%)	0(0.0%)	3(60.0%)
	Discreet	0(0.0%)	0(0.0%)	4(80.0%)	0(0.0%)	0(0.0%)	3(60.0%)	0(0.0%)	1(20.0%)	1(20.0%)	0(0.0%)	0(0.0%)	0(0.0%)	0(0.0%)	3(60.0%)	0(0.0%)
	Moderate	3(60.0%)	2(40.0%)	0(0.0%)	0(0.0%)	0(0.0%)	0(0.0%)	2(50.0%)	0(0.0%)	2(40.0%)	0(0.0%)	0(0.0%)	2(40.0%)	0(0.0%)	0(0.0%)	0(0.0%)
	Intense	2(40.0%)	3(60.0%)	0(0.0%)	5(100%)	0(0.0%)	0(0.0%)	2(50.0%)	4(80.0%)	0(0.0%)	4(80.0%)	4(80.0%)	2(40.0%)	5(100%)	2(40.0%)	1(40.0%)
Characteristics of the infiltrator	Absent	0(0.0%)	0(0.0%)	0(0.0%)	0(0.0%)	4(100%)	0(0.0%)	0(0.0%)	0(0.0%)	2(40.0%)	0(0.0%)	1(20.0%)	1(20.0%)	0(0.0%)	0(0.0%)	3(60.0%)
	PMN	5 (100%)	3(60.0%)	0(0.0%)	5(100%)	0(0.0%)	0(0.0%)	5(100%)	4(80.0%)	1(20.0%)	5(100%)	0(0.0%)	0(0.0%)	5(100%)	1(20.0%)	0(0.0%)
	MMN	0(0.0%)	2(40.0%)	5 (100%)	0(0.0%)	0(0.0%)	5(100%)	0(0.0%)	1(20.0%)	2(40.0%)	0(0.0%)	4(80.0%)	4(80.0%)	0(0.0%)	4(80.0%)	2(40.0%)
Cellularity	Absent	0(0.0%)	0(0.0%)	0(0.0%)	5(100%)	0(0.0%)	0(0.0%)	0(0.0%)	0(0.0%)	0(0.0%)	4(80.0%)	4(80.0%)	3(60.0%)	5(100%)	2(40.0%)	1(20.0%)
	Discreet	5(100%)	3(60.0%)	0(0.0%)	0(0.0%)	0(0.0%)	0(0.0%)	4(100%)	3(60.0%)	1(20.0%)	1(20.0%)	0(0.0%)	0(0.0%)	0(0.0%)	0(0.0%)	1(20.0%)
	Moderate	0(0.0%)	2(40.0%)	3(60.0%)	0(0.0%)	4(100%)	3(60.0%)	0(0.0%)	1(20.0%)	2(40.0%)	0(0.0%)	0(0.0%)	0(0.0%)	0(0.0%)	2(40.0%)	1(20.0%)
	Intense	0(0.0%)	0(0.0%)	2(40.0%)	0(0.0%)	0(0.0%)	2(40.0%)	0(0.0%)	1(20.0%)	2(40.0%)	0(0.0%)	1(20.0%)	2(40.0%)	0(0.0%)	1(20.0%)	2(40.0%)
Re-epithelialization	Absent	5(100%)	3(60.0%)	0(0.0%)	5(100%)	0(0.0%)	0(0.0%)	2(50.0%)	0(0.0%)	0(0.0%)	3(60.0%)	2(40.0%)	2(40.0%)	5(100%)	1(20.0%)	1(20.0%)
	Partial	0(0.0%)	2(40.0%)	0(0.0%)	0(0.0%)	0(0.0%)	0(0.0%)	2(50.0%)	2(20.0%)	0(0.0%)	2(40.0%)	2(40.0%)	0(0.0%)	0(0.0%)	1(20.0%)	0(0.0%)
	CDis	0(0.0%)	0(0.0%)	2(40.0%)	0(0.0%)	0(0.0%)	0(0.0%)	0(0.0%)	1(20.0%)	3(60.0%)	0(0.0%)	0(0.0%)	0(0.0%)	0(0.0%)	0(0.0%)	0(0.0%)
	COrg	0(0.0%)	0(0.0%)	3(60.0%)	0(0.0%)	4(100%)	5(100%)	0(0.0%)	2(40.0%)	2(40.0%)	0(0.0%)	1(20.0%)	3(60.0%)	0(0.0%)	3(60.0%)	4(80.0%)

Descriptive analysis (percentage). CDis: complete disorganized; COrg: complete organized; PMN: polymorphonuclear; MNN: monomorphonuclear. Source: own authorship.

**Figure 3:**
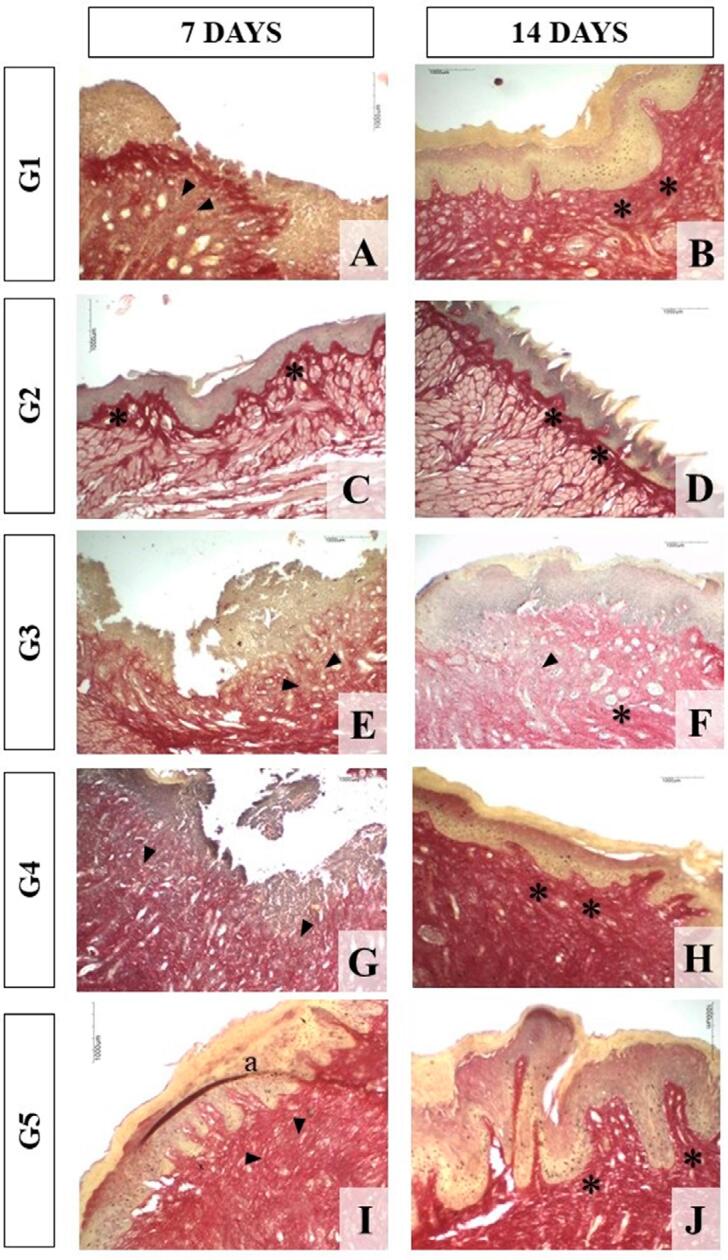
Morphological analysis of the experimental groups. Representative photomicrographs of histological sections (Red Sirius) to illustrate collagen expression on the 7th and 14th days obtained using a 10× objective. The groups showed a similar pattern from the 7th to the 14th day, in which fibers showed a normal appearance and a more delicate pattern (arrowhead), except for G2, which exhibited thicker but disorganized collagen fibers (asterisk). On day 14, the collagen fibers were thicker and organized in all groups. The G2 manifests a pattern close to normality. a: technique artifact.

According to the morphological analysis of the total collagen deposition pattern from histological sections stained with Sirius Red, on the 3^rd^ day, the groups approached the disorganized collagen pattern with delicate thickness and an increased deposition depth, except for the group subjected to the Fluid Extract (G2), which exhibited a disorganized deposition pattern with thick fibers. As the experiment progressed, all groups demonstrated a similar pattern of thick and disorganized fibers on the 7^th^ day. However, the depth from the basal epithelial layer to the muscle tissue increased in G1, G3, and G4 on the 7^th^ day, with only G3 maintained the increment till the 14^th^ day. On day 14, the fibers in all groups exhibited a thick homogeneous appearance with disorganized bundles, except for G2, which exhibited a thick homogeneous pattern with organized bundles and depth from the basal layer to muscle tissue with normal thickness. Figure 3 illustrates the main morphological characteristics of collagen patterns in the experimental groups.

### Histomorphometric analysis

**Figure 4:**
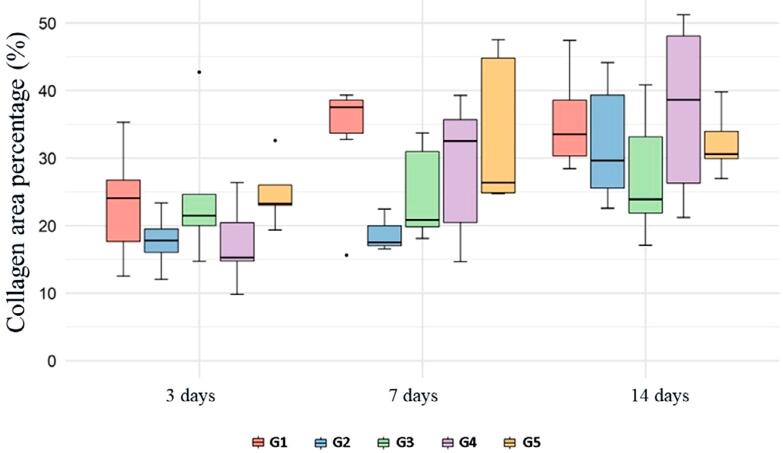
Box plot of percentage of collagen area as a function of group and time

Inter-rater reproducibility analysis was performed to analyze the percentage of collagen area in sections stained with Sirius Red. The results of this quantitative evaluation using intraclass correlation were considered satisfactory (ICC=0.47). Table 4 and Figure 4 show the data obtained from the quantitative analysis of the collagen area. In this evaluation, only the group submitted to exclusive aPDT (G4) showed a significant increase in this percentage on day 7 of treatment compared with day 3 (*p*<0.05). However, when comparing the collagen area on day 14 with that of day 3, it showed a significant increase in groups G1, G2, and G4 (*p*<0.05). In the comparative analysis between the groups, on day 7, G2 showed a smaller percentage of collagen area than G1 and G5 (*p*<0.05). However, on day 14 the area of collagen showed no significant difference between the groups (*p*>0.05).

**Table 4:** Mean (standard deviation) of the percentage of collagen area as a function of group and time (LBO/UFBA, 2022)

Group	Time (days)		
	3	7	14
G1	23.17 (8.17)^Ba^	33.56 (9.11)^ABa^	35.39 (7.18)^Aa^
G2	17.74 (4.63)^Ba^	18.83 (3.18)^Bb^	32.12 (8.98)^Aa^
G3	24.70 (10.68)^Aa^	24.68 (7.12)^Aab^	27.36 (9.53)^Aa^
G4	17.33 (6.29)^Ba^	28.51 (10.48)^Aab^	37.07 (13.15)^Aa^
G5	24.85 (4.94)^Aa^	33.65 (11.46)^Aa^	32.24 (4.90)^Aa^

Distinct letters (uppercase horizontally and lowercase vertically) indicate statistically significant differences (p≤0.05). p(group)=0.0444; p(time)<0.0001; p(interaction)=0.0906. Analysis performed with generalized linear models. Source: own authorship.

## Discussion

Ulcerated lesions in the oral mucosa cause pain and discomfort, leading to difficulties in eating, speaking, and swallowing.^23,24^ Some individuals may experience a delay in the healing process due to the nature of the condition or the oral environment, which is rich in microorganisms that can contribute to wound contamination.^25-28^ This justifies the search for treatment modalities that can accelerate and improve this process. This study aimed to evaluate the effect of chamomile with or without aPDT on the healing time of mechanically induced ulcers on the dorsum of the tongue of rats in different presentations to facilitate the modulation of tissue repair using therapies with no contraindications when combined with other therapeutic agents and minimal unwanted effects. The justification lies in chamomile having proven efficacy in tissue repair in the oral cavity^4,25,29-32^ but being an alternative used empirically; therefore, it should be clinically tested in its various forms. The novelty of this study is due to chamomile being included as an infusion as a therapeutic option alone or associated with aPDT.

The aPDT is inferred to favor wound healing by eliminating local microorganisms; however, the results are contradictory.^16,18,33,34^ In this study, the combination of chamomile and aPDT could not accelerate tissue repair in the rats’ tongues, both from clinical and histological parameters. However, when analyzed in isolation, clinically, aPDT greatly reduced the lesion in the initial phase of the repair. However, Chamomile fluid extract positively modulated all phases of healing, clinically and histologically, being the agent that exhibited more linear and satisfactory results.

Based on the clinical measurements, the aPDT group (G4) presented a similar clinical reduction, with statistical significance similar to those of the other groups. From this period onward, the groups were equivalent in this aspect, except for G1. Initially, aPDT promoted a more accelerated scar repair; however, the other groups achieved the same objective as the experiment progressed. These results are consistent with those of Sperandio, et al.^18^ (2010) and Zuhayri, et al.^34^ (2022). Despite methodological differences, at the end of the study (day 14), all groups had clinically healed skin wounds, as in our study.

Deyhimi, et al.^16^ (2016) also conducted an experimental study on animals and obtained different results from this study, clinically. An ulcer was induced using a punch of 2 mm diameter in the jugal mucosa of 48 male *Wistar* rats, divided into three groups: Control, Laser, and aPDT. The aPDT group received this therapy on the wound for 10 s at a single point, between days 1–4 and 6–9 of the study, which totaled 8 applications. The authors clinically observed a delay in the healing of ulcers in the aPDT group, suggesting a possible inhibitory effect due to its inferior results compared with the group with no treatment modality.

When evaluating the total proportion of animals with ulcers in each group during the experiment, on day 8, only the animals in the groups subjected to the two different chamomile formulations (G2 and G3) exhibited a more accelerated clinical reduction of ulcers, with complete regression in all animals on day 9 of the experiment. This finding is consistent with those of previous studies, which reported the ability of chamomile to accelerate healing in the oral cavity compared with the group that received no therapy^1^ or even those that received topical corticosteroids.^4,25^ In a study by Martins, et al.^4^ (2009), *Wistar* rats underwent traumatic tongue ulceration using a 3 mm diameter punch. The animals received daily applications of the fluid extract of chamomile Ad-muc^®^ or topical corticosteroids. All animals in chamomile group showed complete wound healing on day 5 of the experiment, 9 days before the other groups. A possible reason for the faster clinical repair compared with our study is that the injury was induced with a smaller-diameter scalpel punch. Regardless of this methodological difference, chamomile in the different presentations accelerated ulcer healing in the oral cavity.

Morphological analysis according to the groups and periods of euthanasia showed that all therapies behaved predictably at the 3^rd^ day, demonstrating low cellularity, incomplete re-epithelialization, high intensity of edema and acute inflammatory infiltrate, and a predominance of the polymorphonuclear cells. This finding is consistent with those of previous studies evaluating chamomile’s effect on tissue repair.^1,4,25^ Martins, et al.^4^ (2009) observed that both the group that received a fluid extract of chamomile Ad-muc^®^ and the control group showed no re-epithelialization in all samples, and the connective tissue exhibited moderate to intense amounts of inflammatory cells. Duarte, et al.^1^ (2011) also performed a morphological analysis and observed a wide ulcerated area and intense acute inflammation with a predominance of polymorphonuclear cells after 3 days in both groups. This study also verified intense acute inflammation with a predominance of polymorphonuclear cells during the experimental phase.

Regarding the analysis of the same morphological variables in the initial phase of repair in studies involving aPDT in the oral cavity^16^ and skin wounds^18^ in animals, the results are consistent with the positive modulation of the inflammatory phase of this process. Deyhimi, et al.^16^ (2016) verified that on day 2 of the experiment, 50% of the sections in the aPDT group showed a moderate amount of collagen, the beginning of the granulation tissue formation, and re-epithelialization, compatible with moderate healing. On day 4, the groups were equal, with a slight superiority in the aPDT group. Similarly, Sperandio, et al.^18^ (2010) observed that after 3 days of study, re-epithelialization in cutaneous tissue was more advanced in the groups subjected to Laser and aPDT, and inflammatory infiltrates were significantly lower in both experimental groups. These findings are inconsistent with those of our study, in which the photodynamic action in G4 and G5 could not reduce the edema, and the positive modulation of the repair was subtle. In these groups, tissue biostimulation to accelerate re-epithelialization was discreet on day 3 of the experiment.

Regarding the 7^th^ day of the study, the chamomile fluid extract stood out in the analyses of the morphological variables, with superior results, which, despite having obtained fluctuating indices, maintained a similar pattern with each other. This finding is consistent with those of previous studies that adopted the use of chamomile fluid extract every 12 h,^1,4^ according to the manufacturer’s instructions.^20^ Duarte, et al.^1^ (2011) observed at 7 days that although the animals in the control and chamomile fluid extract groups showed the formation of a new thin orthokeratinized stratified squamous epithelium, no edema, and moderate mononuclear cells, the fibroblasts were arranged in a less organized manner in the samples of the control group. The results of this study, combined with those of our study, strengthen the theory that phytotherapy validates the pharmacological effect of plants that have undergone careful constituents’ inspection and controlled studies,^10,30^ which makes them suitable for treating several diseases, according to the National Health Surveillance Agency in Brazil.^30^ Notably, the chamomile infusion group did not show results similar to those of the fluid extract group.

At the end of the experiment, the group that received a fluid extract of chamomile (G2) behaved similarly till day 7, demonstrating superior results for all histomorphological variables. Furthermore, this group was the only one that presented 100% of the sections with complete and organized re-epithelialization associated with the formation of new lingual papillae, which denotes an advanced degree of differentiation compared with the other groups. These results are consistent with those of previous studies,^1,4,25^ particularly the study of Duarte, et al.^1^ (2011), who noticed the differentiation of the lingual papillae in a more pronounced way in the samples of the animals treated with chamomile from the 10^th^ day.

In the same period, the control group (G1) and the chamomile infusion group (G3), exhibited histological behaviors compatible with the natural process of tissue repair, as described in the literature.^4,35^ This result is likely due to the final concentration of chamomile used in the infusion. The preparation method followed the standards proposed by the last phytotherapy form published by the National Health Surveillance Agency from Brazil^36^ in 2021 and by the European Medicines Agency (EMA) in 2015;^37^ however, the concentration of chamomile in the ready-made infusion could not be obtained. Therefore, despite being indicated for cure or relief of illnesses using the plant species in its raw form, these plants do not undergo quality control using clinical tests. Thus, herbal medicine, including medicinal plants, stands out for presenting strict control in its formulation. However, indicating that regulatory agencies refrain from contraindicating the use of medicinal plants in their raw or commercial form since they have been traditionally used for centuries in a way that benefits humanity is urgent.^38^ In Brazil, which has a rich and diverse flora, raw materials are highly available, which reduces the cost and facilitates access by the population.^10^ Furthermore, new studies addressing the use of chamomile as an infusion at different concentrations are essential for validating therapy based on the use of this natural agent.

Another important factor that should be highlighted is that infusion chamomile is presented in a liquid form, whereas the fluid extract is close to the mucoadhesive formulation, which provides greater adherence to the tissues and thus remains longer in place, increasing the action of the therapeutic agent.^38^ Possible reasons that exemplify these findings and influence the therapeutic action of chamomile include the way the plant was manipulated or processed, the form and frequency of application, the concentration, and the vehicle used.^1,31,32^

At the end of the experiment, the groups subjected to aPDT (G4 and G5) showed unfavorable results since they were the only groups that did not present all the sections re-epithelialized but presented infiltration and intense edema in part of the sections in the same period. Regarding the fibroblastic population, G4 was the only group that exhibited a greater number of sections without cellularity compatible with forming a new collagenous matrix. These findings may be associated with the mechanism of action of aPDT, which primarily aims to eradicate microorganisms^11,12^ and has no confirmed action in tissue repair. This action is based on the destruction of microorganisms depending on the type of cell treated, the concentration of the photosensitizer used, and the dose of light energy provided, with high doses tending to promote cell death by necrosis, whereas lower energy doses cause death via apoptosis^11^. Therefore, we assume that this study adopted a protocol in which the dosimetry was not elevated since Sperandio, et al.^18^ (2010) used this same parameter in a previous study and achieved therapeutic success. However, when comparing the frequency of aPDT application between the two experiments, a single aPDT was applied in the pioneering study,^18^ whereas this study used the resource on alternate days, totaling eight sessions, which does not seem to have accelerated the repair process. Deyhimi, et al.^16^ (2016) also performed eight sessions of aPDT for 14 days. The established protocol was different from that of our study in terms of dosimetry, pre-radiation time, and application interval. However, the results were similar in that the tissue repair was not accelerated. Therefore, the authors^16^ recommend using this therapeutic modality in the first stages of wound repair to promote the initial antimicrobial effect, preventing the perpetuation of redox reactions and not delaying tissue healing.

In addition to performing a single session of aPDT with the same dosimetry at the beginning of the experiment, the authors of this study suggest subsequent sessions of laser photobiomodulation on alternate days, with dosimetry capable of accelerating healing, which is already well-established in the literature.^39^ Furthermore, in infectious lesions, such as herpetic lesions^13^ and those resulting from severe acute respiratory syndrome coronavirus 2 (SARS-CoV-2)^40^ or even ulcers with inflammatory origin as a result of antineoplastic therapy, but which easily become infected, as seen in the case with mucositis,^25^ combining aPDT with laser photobiomodulation is already a reality, with an increasing number of studies using this approach.^13,17,40^ Notably, in our study, the ulcers were not intentionally contaminated; these lesions were exposed to the local microbial flora characteristic of the oral cavity, thus the antimicrobial potential of aPDT was not analyzed.

Another highlight of aPDT success is the proper interaction between the light source and the photosensitizer. Regarding the light source, the type of light used and the parameters, including power, fluence, dose per point, total dose, and application time, should be considered. Laser in the red wavelength has been highlighted in research^13^ since most coloring substances are activated at this wavelength, 630–700 nm, promoting the antimicrobial effect and favoring tissue repair.^33^ These findings reiterate that the dosimetry parameters and the photosensitizer dye adopted in this study are adequate for favorable results.

Regarding the morphological analysis of the total collagen deposition pattern using histological sections stained with Sirius Red, from the beginning to the end of the experiment, the groups exhibited a regular pattern compatible with the natural course of the tissue repair process,^35^ in which disorganized collagen deposition was observed in the inflammatory phase, with delicate thickness and increased depth. Gradually, these fibers thickened in the proliferative phase and remained disorganized until the end of the experiment. However, the G2 group exhibited a differentiated pattern of collagen fibers. From day 3 onwards, a disorganized deposition was observed with thick fibers. On day 14, in addition to a pattern of homogeneous collagen thickness in the sections, we observed an organized deposition and a pattern close to normality. Similarly, in the study by Duarte, et al.^1^ (2011), the histomorphological analysis showed the organized disposition of the fibroblasts and the collagen fibers parallel to the surface in the tissue repair of rats treated with the fluid extract of chamomile, from day 7 to 14 of the experiment.

Regarding the histomorphometric analysis of the percentage of collagen areas, the G4 demonstrated a significant increase on days 7 and 14 of treatment than on day 3. These findings suggest the possibility of aPDT as a biostimulator in the underlying connective tissue, where the collagen area increased due to the increase in fibroblastic activity, favoring tissue repair modulation. The study by Zuhayri, et al.^34^ (2022) reached a similar finding, with an *in vivo* analysis of mice subjected to aPDT for cutaneous ulcers showing a greater deposition of type I collagen fibers and elastic fibers than the other groups, from days 7–14 of the experiment. In contrast, Deyhimi, et al.^16^ (2011) did not achieve results using the semi-quantitative analysis of collagen deposition compatible with the findings of Zuhayri, et al.^34^ (2022) and of our study. In the latter case, aPDT initially seemed to satisfactorily modulate the tissue repair, and was the only group that exhibited abundant collagen fiber content. However, the control group showed better outcomes than the aPDT group throughout the experiment. Therefore, we suggest conducting studies with a longer period of analysis to allow the evaluation of the tissue repair remodeling stage and a more reliable analysis of the deposition and organization pattern of collagen fibers.

Studies involving other aPDT protocols and chamomile formulations in animal models and randomized clinical trial studies on humans are recommended to maximize and confirm the results obtained. Furthermore, additional analyses of tissue samples using immunohistochemistry, fluorescence microscopy, and transmission electronics are also recommended, since those are methods described in the literature for tissue repair.^22,34^ Notably, the analyses performed in this study allowed to investigate these proposed treatment modalities.

## Conclusion

Clinically, all the evaluated groups showed healing of the wound on the dorsum of the tongue. Regarding the morphological and morphometric parameters associated with the epithelial tissue, different formulations with chamomile positively modulated tissue repair patterns clinically, and the fluid extract of chamomile exhibited better histological patterns of tissue repair than the other groups in terms of organization and quantity of collagen deposition tissue.
